# Naview: A d3.js Based JavaScript Library for Drawing and Annotating Voltage-Gated Sodium Channels Membrane Diagrams

**DOI:** 10.3389/fbinf.2022.774417

**Published:** 2022-02-11

**Authors:** Marcelo Querino Lima Afonso, Néli José da Fonseca Júnior, Thainá Godinho Miranda, Lucas Bleicher

**Affiliations:** ^1^ Departamento de Bioquímica e Imunologia, Instituto de Ciências Biológicas, Universidade Federal de Minas Gerais, Belo Horizonte, Brazil; ^2^ Cellular Structure and 3D Bioimaging, European Molecular Biology Laboratory, European Bioinformatics Institute, Hinxton, United Kingdom

**Keywords:** membrane plot, voltage gated sodium channel (NaV), d3.js, data visualization, javascript

## Abstract

Voltage-gated sodium channels (Nav) are membrane proteins essential to initiating and propagating action potential in neurons and other excitable cells. For a given organism there are often multiple, specialized sodium channels found in different tissues, whose mutations can cause deleterious effects observed in numerous diseases. Consequently, there is high medical and pharmacological interest in these proteins. Scientific literature often uses membrane diagrams to depict important patterns in these channels including the six transmembrane segments (S1–S6) present in four different homologous domains (D1–D4), the S4 voltage sensors, the pore-lining residue segments and the ion selectivity filter residues, glycosylation and phosphorylation residues, toxin binding sites and the inactivation loop, among others. Most of these diagrams are illustrated either digitally or by hand and programs specifically dedicated to the interactive and data-friendly generation of such visualizations are scarce or non-existing. This paper describes Naview, an open-source javascript visualization compatible with modern web browsers for the dynamic drawing and annotation of voltage-gated sodium channels membrane diagrams based on the D3.js library. By using a graphical user interface and combining user-defined annotations with optional UniProt code as inputs, Naview allows the creation and customization of membrane diagrams. In this interface, a user can also map and display important sodium channel properties, residues, regions and their relationships through symbols, colors, and edge connections. Such features can facilitate data exploration and provide fast, high-quality publication-ready graphics for this highly active area of research.

## Introduction

Voltage-gated sodium (Na+) channels are key signaling membrane proteins responsible for electrical excitability, also involved in biological processes in non-excitable cells, and of considerable physiological and pharmacological interest (Cardoso and Lewis, 2018). Voltage-gated Na + channels (Navs) can generate and propagate action potentials in excitable cells due to channel opening and fast inactivation mechanisms that regulate the permeation of Na + ions across the membrane (Capes et al., 2012; Xia et al., 2013; Kubota et al., 2017). These channels are present in a large variety of organisms, the domain architecture of human Navs being observed in all animals. Their dysfunction is involved in severe diseases such as epileptic seizures, migraines cardiac arrhythmias, as well as pain-related neuropathies (Xia et al., 2013; Erickson et al., 2018). Sodium channels are involved in multiple physiological roles within a given organism, including the transmission of somatosensory signals, angiogenesis, muscle contraction, and immune cell maturation (Cardoso and Lewis, 2018). In addition, insect sodium channels are potential targets for both natural and synthetic insecticides and are therefore of agricultural interest (Zhang et al., 2016).

Each channel consists of an alpha subunit and auxiliary beta subunits that modify the properties of the first (Widmark et al., 2011). The alpha subunit is composed of a single chain of four sub-units in tandem (Domains I-IV), each formed by a structure of six transmembrane helices (6TM, H1-H6) that associate as tetramers to form a channel. Small extracellular and intracellular loops connect each helix, and the pore loops and large intracellular loops connect each domain (Yu and Catterall, 2003). In mammals, nine isoforms of these channels are found (Gene names SCN1A-SCN11A) possessing different functional roles, properties, and tissue-specific distributions among cells of the central and peripheral nervous systems (Chowdhury and Chanda, 2019). Post-translational modifications such as glycosylations and phosphorylations are part of the cellular modulation repertoire of these channels *in vivo*, being mostly found within the intracellular loop between the first and second domain of these channels (Scheuer, 2011; Laedermann et al., 2015; Cardoso and Lewis, 2018).

Graphical representations of the Nav alpha subunit transmembrane architecture are widely used in the scientific literature, with the earliest examples dated from the late 1980s—(Tanabe et al., 1988; Trimmer et al., 1989; Chiamvimonvat et al., 1996; Marban et al., 1998; Yu and Catterall, 2003; Yamaoka et al., 2006; Wood and Iseppon, 2018; Zybura et al., 2021). In these diagrams, membranes are disposed as either boxes or a bilayer lipid, transmembrane helices are shown as rectangles or cylinders, and loops as curved lines. Features commonly described by such plots include the voltage sensing helix S4, the fast inactivation motif IFM, glycosylation and phosphorylation sites, drug binding sites, important mutation sites, relevant sites for subunit interaction, and toxin binding sites. These features are usually displayed as either text or symbols inside the diagram.

Although sodium channel diagrams have been used for over 30 years, the availability of tools dedicated to an automated generation of such plots has been limited, but options for simpler diagrams with varying features are available. TOPO2 (Johns, 2010) reads an input indicating the number of segments in a protein chain, start/finish residues for transmembrane or partially inserted segments and residues to be colored and generates a simplified color diagram. Topology diagrams can also be drawn by using the output of a topology detection software such as HERA (Hutchinson and Thornton, 1990) and feeding it to topology drawing software such as TopDraw (Bond, 2003). This approach can also be used for globular proteins, but does not allow for individual residue/segment annotation, and includes no information about membrane insertion, being restricted to the secondary structure topology obtained from a PDB file. Membrane diagrams with individual annotations can be created using TMRPres2D (Spyropoulos et al., 2004) using user-provided info or importing information about transmembrane boundaries using public databases. The LaTeX based Protter web application (Omasits et al., 2014) and Textopo (Beitz, 2000) are capable of generating membrane protein diagrams in which each residue is displayed as geometric forms (often as circles). Whereas annotations can be easily included in both programs as symbols, text or specific colors, secondary structures cannot be easily distinguished in the diagrams of Protter and Textopo.

Various commercial and open source alternatives dedicated to drawing chemical compounds such as MarvinSketch, ChemDoodle, BKchem, XDrawChem, JChemPaint, ACD/ChemSketch, and MolView often have modules dedicated to the 3D visualization of proteins, but generating 2D diagrams (Krause et al., 2000; Todsen, 2014; Bergwerf, 2015). ChemDraw is one of the few alternatives including the possibility of drawing such diagrams in a highly dynamic and easy-to-use interface but lacking the possibility of direct inclusion of protein related data (Cousins, 2005).

Sodium channels membrane diagrams remain popular despite the increasing deposition of Nav structures in the last years, especially by cryogenic electron microscopy (Ahuja et al., 2015; Pan et al., 2018; Xu et al., 2019; Jiang et al., 2021), and the vast number of software dedicated to the 3D visualization of protein molecules such as PyMOL (Schrödinger, 2015), UCSF Chimera (Pettersen et al., 2004), VMD (Humphrey et al., 1996), Jmol (Jmol development team, 2016), and JavaScript based tools such as 3Dmol (Rego and Koes 2015), iCn3D (Wang et al., 2020), Litemol (Sehnal et al., 2017), NGL Viewer (Rose and Hildebrand 2015) and Mol* (Sehnal et al., 2021). Often used alongside figures rendered from 3D structures, the persistent usage of Nav diagrams could be attributed to their summarizing capacity. The alpha subunit of Navs often possess a length of more than 1,500 amino acids which can be challenging to depict when their complex topology is taken into account: four domains of six transmembrane helices and a reentrant loop, long and short interdomain loops disposed on either the intra or extracellular faces of the plasma membrane. Due to this the explicit representation of some features could require multiple 3D poses.

This publication describes Naview, an open-source d3.js based JavaScript library for drawing and annotating voltage-gated sodium channels membrane diagrams. Naview can highlight essential Nav features by using custom data provided by the user to modify the text, color, and connecting lines at specific helix/loop elements or residues.

## Methods

### Implementation

Naview is implemented as an open-source d3.js based JavaScript web component, which can be used by importing its main CDN file (naview.js) into web pages. The complete documentation of each of the library’s 107 functions and eight global variables can be found at: http://bioinfo.icb.ufmg.br/naview/public/docs/index.html. Naview is freely available under the Apache License 2.0. The complete source code and additional information related to library usage can be found at GitHub (https://github.com/marceloqla/NaView/). In addition to the web component, Naview can also be used as a web application (http://bioinfo.icb.ufmg.br/naview), developed in PHP, allowing direct access to any sodium channel available in the UniProtKb. Naview Style Editor is a graphical user interface that allows plot customization, the upload of residue mapped properties and residue/element interactions, and the download of the plot figures as Scalable Vector Graphics (SVG) or Portable Network Graphics (PNG). The styling information can also be exported as a text file that can be reused in new diagrams.

### Data Input and Processing

**FIGURE 1:**
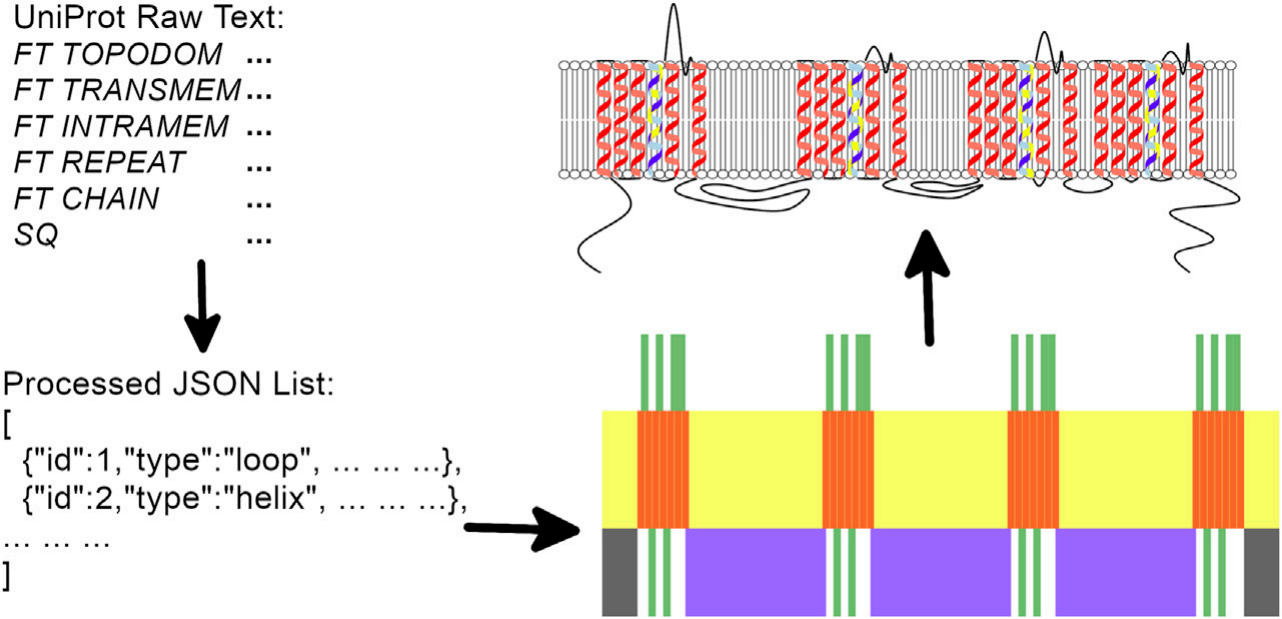
Naview’s general workflow: UniProt formatted data is initially checked and processed before initial draw areas are pre-calculated from the *Style Object* width, height and border definitions. These areas define the positioning of each drawn element in the following order: Membrane, Helices, Short Loops, Pore Loops, Long Loops, N and C terminal Loops. As each protein element is rendered, anchorage points are defined for proper loop positioning. Properties defined from the *Style Obj* or mapped from user selected settings define colors and drawing modes for each of the drawn features.

**FIGURE 2:**
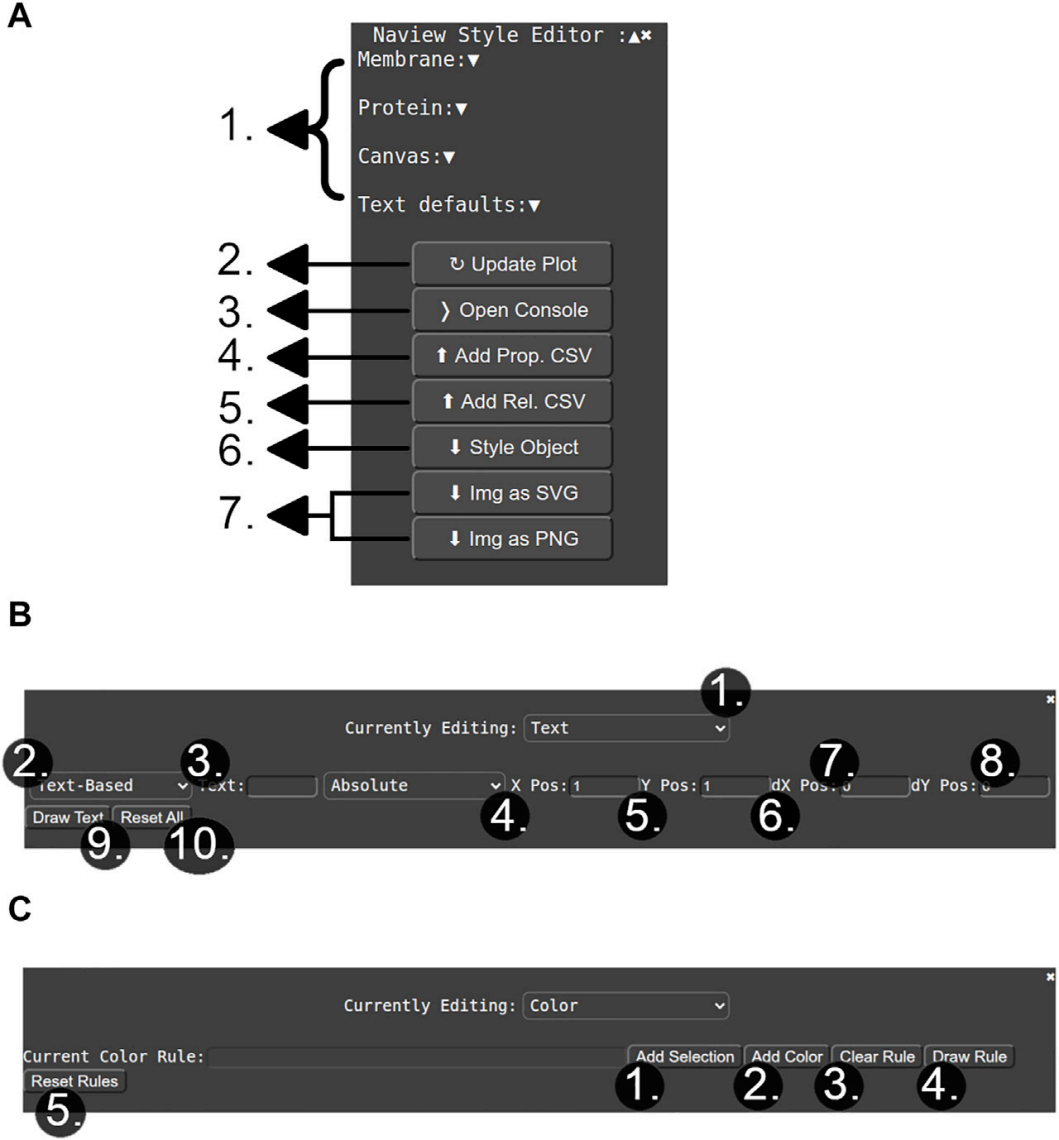
Naview’s style editor. **(A)** Main options of the styling menu including: 1) Dropdowns for options related to each of the drawn diagrams features such as colors, sizes and proportions. 2) Button for refreshing the currently drawn plot. 3) Button for opening the console that allows entering specific text annotation or color rules. 4) and 5) are buttons for adding property and relationship related data to the plot. 6) button for exporting a *Style Object* with the currently selected configurations. 7) Buttons for exporting the plot image in the SVG and PNG formats. **(B)** Console for adding a text annotation or color rule. 1) Dropdown for selecting between the text annotation or color rule modes. 2) Dropdown for selecting the input of free/property based text. 3) Input box for typing the desired text annotation. 4) Text positioning scheme: “absolute” defines text position by the given “x” and “y” 5) and 6) parameters; “relative” defines text position according to a selected element. “dx” and “dy” 7) and 8) shift the text to be drawn in the informed horizontal (“dx”) and vertical direction (“dy”), being especially helpful in the “relative” positioning scheme. 9) Button for appending the currently defined text to the figure. 10) Removes all added text annotations. **(C)** Color rule addition console. 1) Opens a window for allowing specific residue/elements selections. 2) Opens a window for selecting a specific color/property-based color mapping. 3) Clears the currently selected color rule. 4) Updates plot with the currently selected color rule. 5) Removes all previously added color rules.

Two main inputs are generally supplied to Naview for generating a Nav alpha-subunit diagram (Figure 1):

1) A mandatory UniProt formatted text string (hereafter named *Raw Text*) containing the required data plotting a Nav alpha subunit. In the web application version, it is automatically fetched from the UniProtKb, requiring only the sequence identifier;
2) An optional JavaScript Object Notation (JSON) object, hereafter named *Style Object*, containing information related to the elements plot disposition such as their drawing types, widths, heights, scales, and colors (http://bioinfo.icb.ufmg.br/naview/public/docs/symbols/style_obj.html on the documentation for further information on the *Style Object*). When not supplied, a default representation of the *Style Object* is automatically applied. Any drawing options of the *Style Object* can be modified by Naview Style Editor (Figure 2).

Additionally, other inputs related to plotting text, color, and relationship annotations can be supplied. Each of them is described alongside their specific syntax in their dedicated sections.

The UniProt formatted *Raw Text* supplied by the user is then processed for the definition of drawing areas for three possible element types: membrane, helices, and loops which are further sub-divided as short loops, long loops, pore loops, N-terminal loop and C-terminal loop.

### Membrane, Helix and Loop Despictions

The Membrane element can be depicted as a “box” (SVG “rect” element) or as a lipid bilayer (multiple SVG “path” elements). The Style Object controls all specifications of coloring and drawing aspects of these two membrane representations, such as their opacity and relative sizes. Likewise, helices elements can be plotted according to three possible *Style Object* draw types: “box”, “cylinder” and “cartoon”.

**FIGURE 3:**
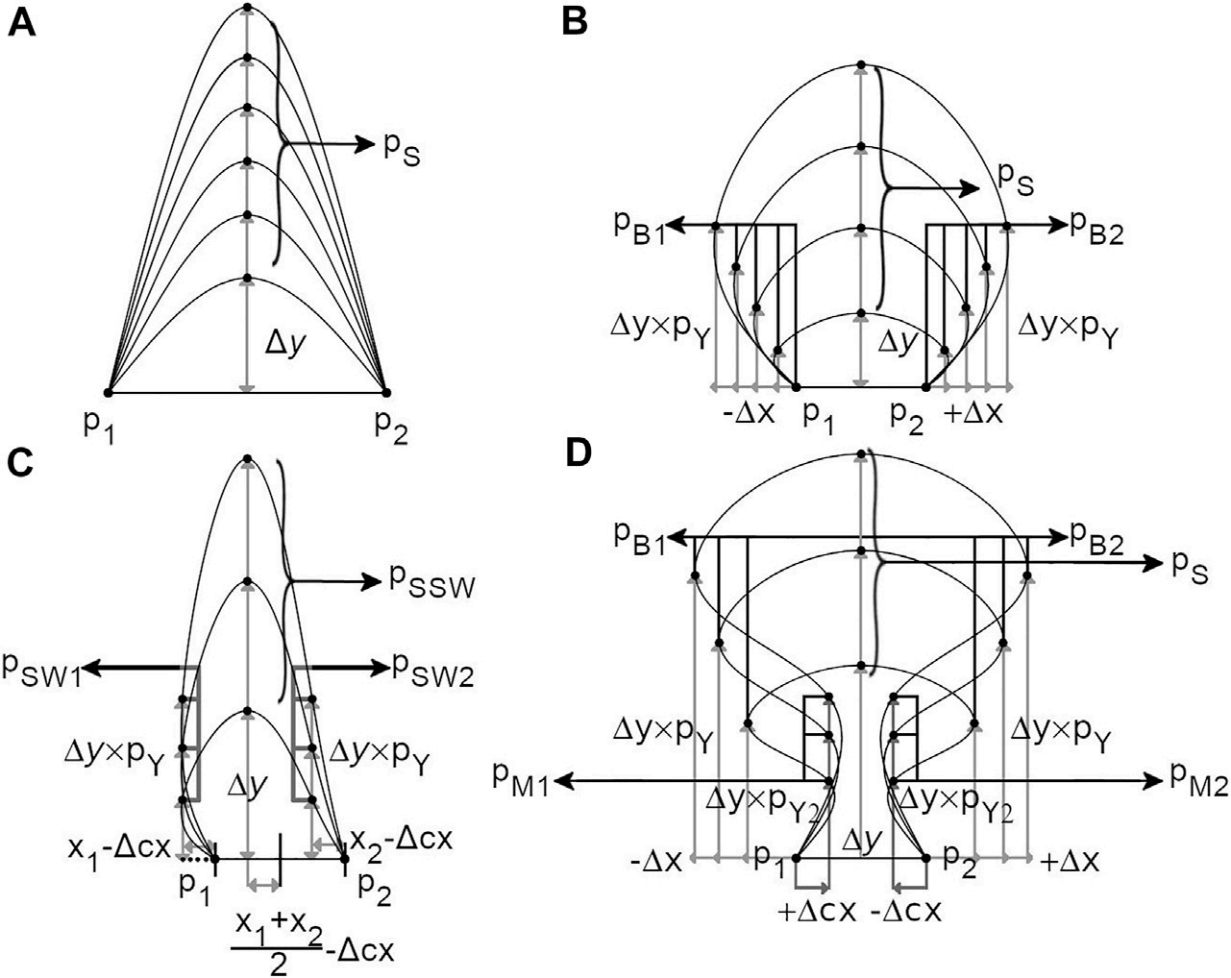
Naview’s curve drawing logic. **(A)** “*Simple*” curve function: a new point p_S_ is generated in the center of two anchoring ponts drawing functions and scaled by a Δy parameter according to the selected loop length scales. **(B)** The “*Bulb*” curve function in which two new points are generated in relation to the “*Simple*” curve type: p_B1_ and p_B1_ whose vertical growth is controlled by p_Y_, a proportion of the total Δy. The horizontal position of these points is given by the Δx parameter in the opposite direction of their closest anchoring points. **(C)** “*Swirl*” curve function is a variation of the “*Bulb*” curve type whose horizontal position is defined in a symetrical direction by a Δcx parameter, defined as a proportion of the distance of the anchoring points to their centroid. **(D)** The “*Mushroom*” curve type includes two new points in relation to the “*Bulb*” curve type: p_M1_ and p_M2_. The vertical position of these points is defined by the p_Y2_ parameter as a proportion of the total Δy, and their horizontal position is defined from the anchoring points positions towards their centroid by the Δcx parameter.

Loops can be drawn by different curves whose rendering depends on their classification. Two aspects are considered for the rendering of these curves: their curve type function and their curve scaling method. Curve type functions describe the shape of a given loop by generating points to be interpolated by the d3.curveNatural function. Distances between these points have fixed or user-selected bounded proportions such that each curve type drawing aspect is scaled according to the Curve Scaling methods defined in the *Style Object*. Curve types common to the short and long loops include the “*Simple*”, “*Bulb*” and “*Mushroom*” curves. The “*swirl*” curve type is specific to short Loops. Pore loops are generated by the “*pore*” curve type and N- and C- terminal by the “*N Curves*” curve type. The availability of multiple curve customization options allows users to customize plot aspects to their preferred style (Figure 3).

Design decisions for the representation of membranes, helices and loops attempted to cover most previously published Nav diagrams (Tanabe et al., 1988; Trimmer et al., 1989; Chiamvimonvat et al., 1996; Marban et al., 1998; Yu and Catterall, 2003; Yamaoka et al., 2006; Wood and Iseppon, 2018; Zybura et al., 2021). The usage of individual elements inside a SVG document for each of the single Nav main secondary elements allowed the attribution of precise cartesian coordinates for each individual residue in this document. This enables the proper assignment of any text, color or edge annotations on the plot by the user.

Naview includes four scales to determine the loop length, depending on each loop type:

• “Fixed” in which a box of fixed height (and possibly width for “Bulb” and “Mushroom” curves) is set for determining the interpolating points of all loops of a given type (Short, Long, Pore or N/C terminus Loops).
• “Scaled” in which the height (and possibly width as above) of the boxes set for determining the interpolating points of all loops of a given type (Short, Long or Pore Loops) are set from a linear, power or logarithmic scale of their amino acid numbers up to a maximum box height (and possibly width).
• “Reslen” in which the height (and possibly width) of each box of a loop-type (Short, Long, Pore or N/C terminus Loops) is defined by a specific pixel value.
• “Custom” in which boxes of fixed specific height (and possibly width for “Bulb” and “Mushroom” curves) are set for determining the interpolating points of each loop of a given type (Short, Long Loops).

All helix and loop coloring, opacity, stroke and scaling settings are controlled by properties of the *Style Obj*.

### Input of Residue/Element Mapped Properties and Relationships

**FIGURE 4:**
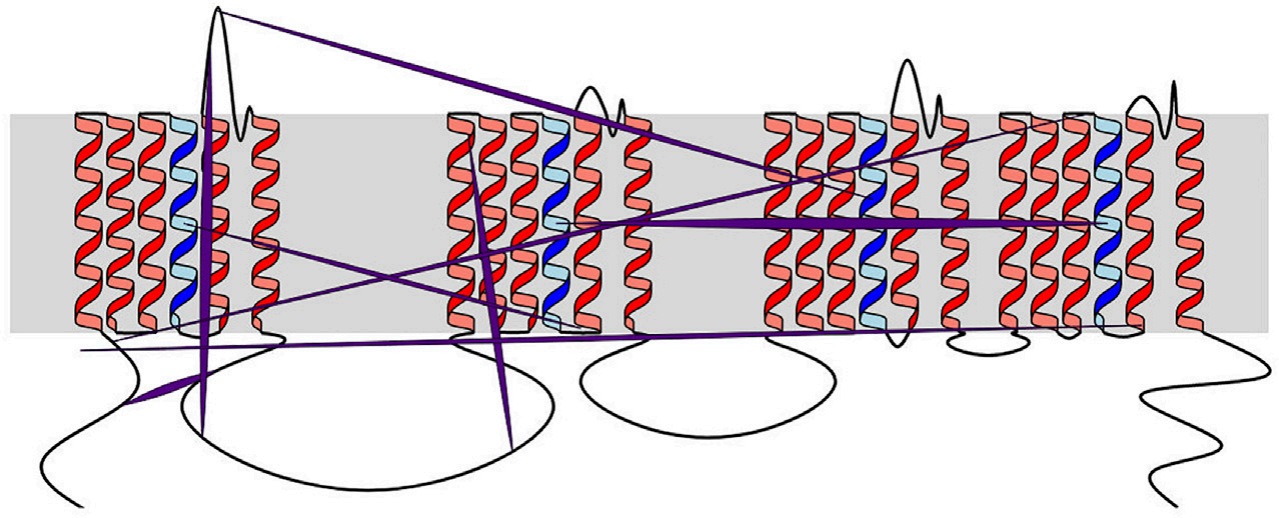
Example of Naview’s relationship drawing. Edges are colored in purple, with their central widths scaled according to the “*raw_weight*” column weights. This scaling allows the visual perception of stronger (larger width) and weaker (thinner width) relationships within the user inputted data. The membrane is shown as a grey box. All helices are shown as red cartoons except for the voltage-sensing helix 4, colored in blue.

**FIGURE 5:**
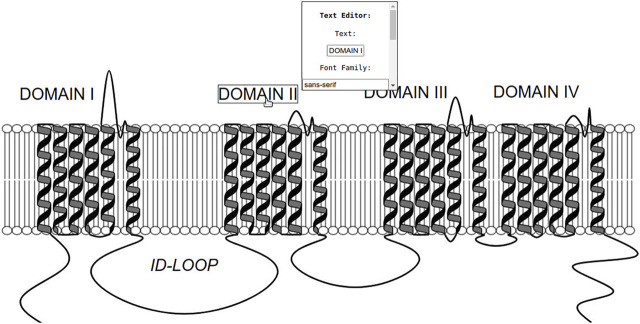
Example of Naview’s text annotations. All domain-indicating texts were added by using the Naview Style Editor console in the text edition mode. Text position can be adjusted by clicking and dragging any added text element. A single click highlights the selected text annotation and allows the editing of its current text and font characteristics. In this example such annotations were used to indicate specific domains (I-IV) and the first intracellular loop (ID-LOOP). Helices are shown as black cartoons and the membrane as a lipid bilayer. All loop residues are scaled to two pixels.

**FIGURE 6:**
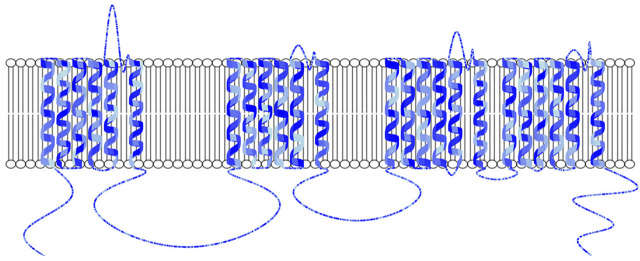
Example of Naview’s property-based color map from lightblue to blue after loading a CSV containing a randomly valued property named “Conservation” ranging from 0 to 1 for each of the protein’s residues. Used color rule: *“ALL, by:Conservation,#ADD8E6;#0000FF, min;max”*. As such residues with a higher “Conservation” value are colored in a darker tone of blue. Helices are shown as cartoons and the membrane as a lipid bilayer. All loop residues are scaled to two pixels.

User-inputted residue properties and residue, helix and loop relationships can also be rendered as text annotations, specific coloring rules and edges between residues or elements (Figures 4–6). The possibility of including properties and relationships in the plot differentiate Naview from drawing-only methods, by allowing the ability of the direct inclusion and visualization of experimental data. Both types of data can be either preloaded alongside the *Raw Text* (Examples in Tables 1 and 2) or included by the Naview Style Editor.

**TABLE 1:** Property table example. First column must be formatted with the “Resid” header followed by digits indicating each residue for a property to be mapped. The following property columns have header strings and are followed by float or integer numbers indicating the value of a property for each residue of a NaV.

Resid	Property
1	0.2871809547
2	0.9835970474
3	0.3891381106
4	0.2391246386

**TABLE 2:** Relationship table example. Four columns are allowed with the following headers: “source”, “target”, “raw_weight”, and “type”. First and second columns indicating the interacting residues or elements. The “raw_weight” column contains an edge weight for color or width mapping. The last column “type” can be used to indicate edge types which can be weighted or colored separately.

Source	Target	Raw_weight	Type
776	660	0.6944505517	Resids
86	469	0.7383026986	Resids
1,308	318	0.4949883823	Resids
305	510	0.9651479396	Resids
1,621	123	0.3030461658	Resids
DomainI; Helix4	DomainII; Loop4	0.08937180957	Elements
DomainII; Helix4	DomainIV; Helix4	0.9300459795	Elements
InterDomain5; Loop	InterDomain1; Loop	0.1476849439	Elements

Specific property values mapped for a set of residues can be loaded and used to generate color scales for differential residue coloring or element mapped text annotations. These properties should be loaded as a JSON object in which each Nav alpha subunit residue index (Example *1,2,3 … 2005* for a Nav containing 2005 residues) is used as a key for another dictionary, whose keys are strings describing a given property and whose values are those of the given properties for the selected residue (Example: *1:{“Conservation”:0.1},2:{“Conservation”:0.3},3:{“Conservation”:0.5}* and henceforth).

Data representing relationships or interactions between different residues/elements present in the plot can be included as a list of JSON inputs in the following format. Example: *{“source”: 1,“target”: DomainI;Helix6, raw_weight:0.5, “type”: “Residue Importance”}*.

### Color Rules and Text Annotations

A list containing multiple color-filling text rules can be loaded as an input for generating a property-based residue color map. Accepted strings for color rules are any residue or element string keys followed by a comma-separated hex or string formatted color. Additionally, when properties have been mapped for a given Nav, they can be used for generating property-specific color maps.

Text annotations can be added as a list of JSON objects containing information about where a specific text should be drawn. This information can either be coded as absolute horizontal and vertical coordinates or as relative coordinates according to the positioning of a given residue or helix/loop element.

Alternatively, both color rules and text annotations can be added by the Naview Style Editor graphical interface (Figures 2, 5, 6).

## Results and Discussion

The existence of a diagram for displaying the alpha-subunit architecture of Nav for over 30 years highlights their usefulness in depicting important properties of these proteins. The Naview d3.js based JavaScript library described in this publication is the first automated method focused on generating these diagrams. Examples and the full documentation for this library can be found at: http://bioinfo.icb.ufmg.br/naview/use and http://bioinfo.icb.ufmg.br/naview/public/docs/index.html.

The construction of transparent, information-rich and thought-provoking visual narratives is an intrinsic challenge in bioinformatics data visualization which requires the management of different graphical elements for efficient communication (Tao et al., 2004; O'Donoghue, 2021). This challenge is addressed by Naview’s through its high customization and data integrative potential and facilitated by the inclusion of a dynamic graphical interface. Since Naview is formatted as a fully documented JavaScript library, its inclusion in web data resources focused on these channels can also be done simply and straightforwardly. By allowing the inclusion of residue mapped properties and relationships, Naview can be used for data exploration and integration purposes beyond the generation of publication-ready Nav figures.

In this publication, we demonstrate Naview and describe the logic of its implementation along with many of its features for plotting text, interactions and color mapped properties of sodium channels. Future updates should be focused on expanding the text annotation syntax to include drawing of polygons, arrows, backgrounds and other symbols, as well as reconfiguring the JavaScript library for drawing schemes and displaying data for any transmembrane/membrane-anchored protein.

## Data Availability

The datasets presented in this study can be found in online repositories. The names of the repository/repositories and accession number(s) can be found in the article/Supplementary Material.
